# Role of prostanoids, nitric oxide and endothelin pathways in pulmonary hypertension due to COPD

**DOI:** 10.3389/fmed.2023.1275684

**Published:** 2023-10-10

**Authors:** Abdullah A. Alqarni, Abdulelah M. Aldhahir, Sara A. Alghamdi, Jaber S. Alqahtani, Rayan A. Siraj, Hassan Alwafi, Abdulkareem A. AlGarni, Mansour S. Majrshi, Saad M. Alshehri, Linhua Pang

**Affiliations:** ^1^Department of Respiratory Therapy, Faculty of Medical Rehabilitation Sciences, King Abdulaziz University, Jeddah, Saudi Arabia; ^2^Respiratory Therapy Unit, King Abdulaziz University Hospital, Jeddah, Saudi Arabia; ^3^Respiratory Therapy Department, Faculty of Applied Medical Sciences, Jazan University, Jazan, Saudi Arabia; ^4^Respiratory Care Department, Al Murjan Hospital, Jeddah, Saudi Arabia; ^5^Department of Respiratory Care, Prince Sultan Military College of Health Sciences, Dammam, Saudi Arabia; ^6^Department of Respiratory Care, College of Applied Medical Sciences, King Faisal University, Al Ahsa, Saudi Arabia; ^7^Faculty of Medicine, Umm Al-Qura University, Mecca, Saudi Arabia; ^8^King Abdulaziz Hospital, The Ministry of National Guard Health Affairs, Al Ahsa, Saudi Arabia; ^9^King Saud bin Abdulaziz University for Health Sciences, College of Applied Medical Sciences, Al Ahsa, Saudi Arabia; ^10^National Heart and Lung Institute, Imperial College London, London, United Kingdom; ^11^Respiratory Medicine, Royal Brompton Hospital, London, United Kingdom; ^12^Department of Respiratory Therapy, King Fahad General Hospital, Jeddah, Saudi Arabia; ^13^Respiratory Medicine Research Group, Academic Unit for Translational Medical Sciences, University of Nottingham School of Medicine, Nottingham, United Kingdom

**Keywords:** nitric oxide, prostanoid, pulmonary hypertension, COPD, endothelin, type 3 pulmonary hypertension, COPD-associated pulmonary hypertension, pulmonary hypertension in COPD

## Abstract

Pulmonary hypertension (PH) due to chronic obstructive pulmonary disease (COPD) is classified as Group 3 PH, with no current proven targeted therapies. Studies suggest that cigarette smoke, the most risk factor for COPD can cause vascular remodelling and eventually PH as a result of dysfunction and proliferation of pulmonary artery smooth muscle cells (PASMCs) and pulmonary artery endothelial cells (PAECs). In addition, hypoxia is a known driver of pulmonary vascular remodelling in COPD, and it is also thought that the presence of hypoxia in patients with COPD may further exaggerate cigarette smoke-induced vascular remodelling; however, the underlying cause is not fully understood. Three main pathways (prostanoids, nitric oxide and endothelin) are currently used as a therapeutic target for the treatment of patients with different groups of PH. However, drugs targeting these three pathways are not approved for patients with COPD-associated PH due to lack of evidence. Thus, this review aims to shed light on the role of impaired prostanoids, nitric oxide and endothelin pathways in cigarette smoke- and hypoxia-induced pulmonary vascular remodelling and also discusses the potential of using these pathways as therapeutic target for patients with PH secondary to COPD.

## 1. Definition, prevalence and treatment of pulmonary hypertension in COPD

Pulmonary hypertension (PH) is a clinical condition defined based on haemodynamic instabilities presented in an increased vascular resistance within pulmonary arteries and is mainly characterised by a mean pulmonary artery pressure (mPAP) that is greater than 20 mmHg and a pulmonary vascular resistance (PVR) greater than 2 Wood Units, as per the new updated ESC/ERS guidelines (1). PH could occur in association with other diseases or isolated for idiopathic purposes, based on which PH is classified (2, 3). This classification divides PH into 5 main groups.

PH due to lung diseases, such as chronic obstructive pulmonary disease (COPD) or interstitial lung disease, is classified as group 3 PH (4). Studies on group 3 PH are not very extensive which makes it an area suitable for further exploration (5–8). COPD is one of the lung diseases that commonly develop into PH (9). Additionally, COPD is a leading cause of morbidity and mortality worldwide and a major cause of disability-adjusted life-years lost worldwide (10, 11). The American Thoracic Society defines COPD as a preventable and treatable disease state characterised by airflow limitation that is not fully reversible, which is progressive and associated with a chronic inflammation response of the lungs to noxious particles or gases (10). COPD is mainly caused by cigarette smoking, but it could also be caused by exposure to biomass fuel combustion, autoimmunity, or chronic infection (10, 12, 13).

Previous studies have reported that the prevalence of PH in patients with COPD varies between 20.5 and 90.8% (14–22). PH is found in advanced COPD, and it is a serious complication involving haemodynamic instabilities leading to frequent exacerbation episodes and decrease in survival rate (5, 9, 23). There is no complete understanding of the pathogenesis of PH in COPD patients; however, studies have shown that PH is characterised in COPD patients by hypertrophy of the pulmonary arterial walls and loss of capillary beds (24). Medium to small pulmonary vasculatures have not been vastly studied in regards of their remodelling. Nevertheless, it is known that such remodelling is linked to maladaptation of endothelial cells to chronic cigarette smoking with increased release of vasoconstrictors and reduced releases of vasodilators and it occurs in all affected pulmonary vessels with varying severities regardless of their size (25).

Hypoxia and inflammation are also drivers of vasculature remodelling and development of PH (26). Due to the damage in alveolar walls caused by alveolar hypoxia, vasoconstriction of the surrounding arteries occurs to maintain ventilation and perfusion balance (8). Consequently, developing PH. However, PH can still occur in COPD patients without hypoxemia (6). Alongside the main symptoms COPD patients experience, which include cough, dyspnea, shortness of breath, and sputum production, the development of PH will cause some haemodynamic symptoms as well (23). Moreover, PH in COPD is known to increase mPAP up to 35 mmHg and higher in some severe cases. Consequently, increasing morbidity and mortality and significantly reducing the quality of life (27).

Together with the treatments of COPD and pulmonary rehabilitation, different COPD-associated PH therapies have been experimented with including supplemental oxygen, calcium channel blockers, statins, and type 1 PH targeted therapies. To date, there has not been any type of therapy to be recognised as the therapy of choice for COPD-associated PH patients. However, some therapies have only presented hemodynamic improvement with inconsistent functional or clinical effects (28). Clinicians have adopted medications approved for group 1 PH, including endothelin receptor antagonist (ERA) and phosphodiesterase-5 inhibitors (PDE5i), nitric oxide, and prostaglandin I_2_ (PGI_2_) analogue, for COPD-associated PH (8, 29). Randomised clinical trials have been done to evaluate the efficacy of group 1 PH therapy on COPD-associated PH patients, most of which were done on PH cases of mild to moderate severity. However, results have not been effective enough as minimal to no effect was seen on exercise capacities as well as limited efficacy on improving the quality of life in such patients (27, 28). Furthermore, hemodynamic status and survival have been found to improve in moderate to severe cases in studies with mixed populations including multiple pulmonary diseases (27, 28). Such results of group 1 PH medications in COPD-associated PH cases are insufficient and conflicting making further exploration of this topic an absolute necessity (6, 27, 28).

## 2. Pathophysiology of COPD-associated pulmonary hypertension

The hallmark of all forms of PH is thought to be pulmonary artery vasoconstriction and remodelling of the pulmonary vessel wall, including dysfunction and proliferation of pulmonary artery cells. Although the pathophysiological mechanisms of COPD in PH are not fully understood, there has been a re-emerging interest in studying the leading causes of PH in COPD with the focus on trying to find the best therapeutic targets for such cases. It is suggested that PH complicating COPD occurs as a consequence of the combined effects of inflammation, cigarette smoke (CS), and hypoxia, which could then lead to elevated PVR and eventually contribute to PH in COPD patients (30). Given that the role of inflammation in inducing pulmonary vascular remodelling has been described elsewhere (31, 32), this review mainly focuses on the roles of hypoxia and CS in the process of pulmonary vascular remodelling in COPD.

### 2.1. Pulmonary vasoconstriction

Pulmonary artery vasoconstriction induced by alveolar hypoxia is a reflex contraction of pulmonary artery smooth muscle cells (PASMCs). This hypoxic pulmonary vasoconstriction is a vasoprotective response to hypoxia to maintain ventilation-perfusion balance, as blood flow in the pulmonary arteries of COPD patients is shifted from hypoxic alveoli toward normoxic alveoli to help minimise ventilation-perfusion mismatch. As the disease progresses, persistent hypoxia (resulting from inadequate delivery of oxygen to the tissues) is thought to lead to elevated PVR and PH in COPD (33).

Over the past two decades, substantially more information has become available and the traditional view that suggests the elevated PVR in COPD can only be a result of hypoxic pulmonary vasoconstriction has been challenged (34, 35). Recent evidence shows that endothelial dysfunction (driven by CS, inflammation, and chronic hypoxia in COPD) also plays a key role in the cause of pulmonary vasoconstriction and pulmonary vascular remodelling in COPD patients (35–38). This suggests that CS can be the primary driver of pulmonary vascular remodelling in COPD (39), although hypoxia is definitely known to induce pulmonary vascular remodelling. Today, the exact mechanistic basis of CS and hypoxia, either individually or in combination, on the induction of pulmonary artery dysfunction and remodelling in COPD is still unclear.

### 2.2. Pulmonary vascular remodelling

#### 2.2.1. Role of pulmonary artery smooth muscle cells

Pulmonary vascular remodelling refers to the key structural changes in the vascular wall that eventually contribute to elevated PAP by increasing PVR in all forms of PH, including COPD-associated PH (40, 41). Pulmonary vascular abnormalities have been attributed to the proliferation of endothelial, smooth muscle, and fibroblast cells, which are cellular components of the three layers of pulmonary vascular wall: intima, media, and adventitia, respectively (42).

The intima is composed predominantly of endothelial cells, and plays an important role in regulating the vascular tone and controlling cell growth. Thickness of the intima layer has been associated with endothelial dysfunction in the pulmonary arteries of COPD patients, which may initiate the process of vascular remodelling (43). In addition, medial wall thickening, caused by proliferative smooth muscle cells, has been observed in patients with COPD (44). More importantly, histochemical and immunohistochemical studies performed in the vasculature of patients with COPD have shown increased number of smooth muscle cells (but not fibroblasts) in the enlarged intima as a result of staining the intimal layer by specific smooth muscle cell and fibroblast markers (31, 41, 43). The proliferation of smooth muscle cells observed in the thickened intima of patients with COPD occurred in an inward direction, thus reducing the vascular lumen (31). This suggests that PASMCs play an important role, in addition to PAECs, in the development of COPD-associated PH.

Although the key signalling pathways involved in the remodelling process are unknown, the pathophysiological changes reported in the innermost two layers of pulmonary artery in COPD, the media and intima, are believed to occur as a result of PASMC proliferation due to PAEC dysfunction (41, 43). Therefore, PASMCs and PAECs are considered the two key cell types that play a major role in the pathophysiology of COPD-associated PH.

#### 2.2.2. Role of pulmonary artery endothelial cells

PAECs are located in the intimal layer of pulmonary arteries that line the vascular lumen and play a key role in controlling cell growth and regulating vascular tone. Apoptosis-resistant and hyper-proliferative PAECs have been reported in patients with group 1 PH (45). It has also been shown that vascular endothelial growth factor (VEGF) receptor inhibitor in combination with chronic hypoxia can cause vascular endothelial cells apoptosis. This initial apoptosis is followed by proliferative vascular endothelial cells in rat models of PH (46), suggesting that the emergence of apoptosis-resistant proliferating vascular endothelial cells may play an important role in vascular remodelling and the development of PH. This is also supported by a more recent study showing that the initial apoptosis, induced by VEGF receptor inhibitor, is followed by increased proliferation of apoptosis-resistant human pulmonary microvascular endothelial cells (47). The accumulative evidence suggests that proliferative PAECs play a role in the pulmonary vascular remodelling in group 1 PH. However, whether PAEC proliferation plays a role in the development of COPD-associated vascular remodelling and PH is largely unknown.

### 2.3. Hypoxia and cigarette smoke

It has been reported that pulmonary vascular remodelling is observed in pulmonary vessels of COPD patients with hypoxemia (48). It has also been suggested that the degree of PH in COPD is directly related to the severity of hypoxemia, and the alteration of pulmonary vessels in COPD at a late stage of the disease is thought to result from chronic hypoxia (44), indicating that hypoxia is an important contributor to vascular remodelling in COPD. This is supported by human and animal cellular experimental studies that demonstrate induction of PASMC proliferation in response to hypoxia. For example, hypoxia has been shown to induce proliferation of human PASMCs (48–50) and rat PASMCs (51). Similarly, experimental studies have also shown that hypoxia can induce proliferation of human PAECs (52, 53). However, there are conflicting data with regards to the direct effect of hypoxia on the proliferation of PAECs in human and animal cellular experimental models. It has been shown that hypoxia alone does not stimulate proliferation of PAECs in human cellular experimental models of hypoxia-induced PH, as well as in hypoxic models of mice and rats (49). These rather contradictory observations may be due to methodological factors such as differences in exposure time and oxygen concentrations. Although the most recent data suggest that hypoxia is a direct stimulus of PASMC and PAEC proliferation, the underlying cause that leads to hypoxia-induced proliferation of PASMCs and PAECs is unclear.

CS is considered the most common etiological factor in developing COPD (54). Although its role in COPD-associated PH is not well characterised, the traditional view that PH in COPD is secondary to chronic hypoxia and emphysema is challenged by clinical and experimental evidence (55). Apparent vascular remodelling has been demonstrated in “healthy” smokers and patients with mild-to-moderate COPD, despite the fact that PH is usually diagnosed in patients with advanced COPD (56). Furthermore, thickness of vascular layers has been observed in patients with COPD and smokers with no sign of airway obstruction (41, 57, 58), suggesting CS plays an important role in vascular remodelling in COPD. These findings are supported by experimental studies showing that CS extract directly stimulates proliferation of human PASMCs (59, 60) as well as PAECs (60), which ultimately contributes to pulmonary vascular remodelling. Interestingly, more prominent remodelling in the pulmonary arteries is present in animals exposed to CS and hypoxia, in comparison to either CS or hypoxia alone (61). These observations support the idea that CS can initiate vascular remodelling, which may be further amplified by chronic hypoxia in advanced COPD. However, the possible contribution of PASMC and PAEC dysfunction in this process remain to be explored.

## 3. Imbalanced vasoactive gene expression and mediator release in COPD-associated pulmonary hypertension

Pulmonary vascular remodelling, as a consequence of PASMC, PAEC, and fibroblasts hypertrophy and proliferation, is an important pathological feature of PH. Current data show that there is significant contribution of PASMC, PAEC, and fibroblasts in the vascular remodelling leading to type 1 PH. However, there has not been enough data showing the contribution of fibroblasts in the vascular remodelling developing COPD-PH (62, 63). Although there is still no specific marker for pulmonary vascular remodelling in all forms of PH, targeting mediators of vascular dysfunction has been shown to be effective in reversing vascular remodelling in group 1 PH. Early evidence suggests that an imbalance of reduced anti-proliferative mediators as opposed to increased proliferative mediators may be critical for the aberrant PASMC proliferation caused by CS and chronic hypoxia in COPD-associated PH (64). However, the extent of the imbalance, the role of the imbalanced individual mediator in vascular remodelling, the contribution of PAECs (particularly PASMCs) in this process, and the effect of CS and hypoxia on the imbalance are still unclear.

### 3.1. eNOS/nitric oxide

Nitric oxide, a potent pulmonary vasodilator, anti-proliferative mediator, and endothelial-1 synthesis inhibitor (65), is generated by nitric oxide synthase (NOS) in vasculature. NOS is known to have three different isoforms named according to their roles: neuronal nitric oxide synthase (nNOS or NOS1), inducible (iNOS) or NOS2, and endothelial NOS (eNOS) or NOS3 (66). nNOS is constitutively expressed in neuronal cells and skeletal muscle, while iNOS is induced at sites of inflammation in response to inflammatory mediators (e.g., TNF-α and IL-1β) in many cell types (67). eNOS is constitutively expressed in vascular endothelial cells and is considered the main source of nitric oxide production in the pulmonary circulation system (68). After being produced by eNOS, nitric oxide is known to activate soluble guanylyl cyclase (sGC), the primary receptor for nitric oxide, resulting in the formation of the second messenger cyclic guanosine monophosphate (cGMP), which causes vasorelaxation and inhibits PASMC proliferation by decreasing the intracellular calcium concentration (56).

Deficiency of eNOS expression has been reported in the pulmonary arteries of smokers (69), in the lung tissues of patients with group 1 PH and non-COPD related PH (70), as well as in lung homogenates of guinea pigs exposed to CS (61). Furthermore, levels of nitric oxide in the plasma of guinea pigs exposed to CS (61) and in CS extract-treated human PAECs (71) are decreased. In addition, hypoxia, a pathological stimulus leading to vascular remodelling in COPD (42), has been shown to reduce eNOS expression and nitric oxide production in the lungs of piglets exposed to chronic hypoxia (72), as well as in human saphenous vein endothelial cells (73). Although there is limited information in the literature concerning the effect of hypoxia and CS on eNOS expression and nitric oxide production in human vascular cells, studies so far suggest an association of reduced nitric oxide bioavailability with PH development and a role for reduced eNOS expression in CS- and hypoxia-induced vascular remodelling. However, the underlying cause and role of CS-induced effects and the synergies between CS and hypoxia in vascular remodelling in COPD remain unclear.

Addressing the nitric oxide bioavailability dysfunction through stimulating sGC together with the inhibition of the cGMP-degrading enzyme phosphosiestrase type 5 (PDE5) can consequently aid in restoring nitric oxide-mediated protective effects in vascular cells which has been widely studied in group 1 PH and has shown anti-proliferative and vasodilatory effects in the pulmonary arteries of patients with group 1 PH (74–76). Recent preclinical evidence showed that treatment with sGC stimulator riociguat for 3 months (following 8 months of CS exposure) can reduce pulmonary vascular remodelling and mean vessel wall thickness, and fully reverse PH in a mouse model of CS-induced PH (77). Similarly, treatment with riociguat has been reported to decrease PVR in retrospective analysis of seven patients with COPD-associated PH (77). These preliminary data, together with the potential effectiveness of inhaled pulmonary vasodilators led to the currently ongoing clinical trial conducted to assess the effect of inhaled sGC stimulator on exercise capacity in COPD-associated PH (78).

In addition to the beneficial effect of sGC stimulator, the use of the PDE5 inhibitor sildenafil was demonstrated to prevent CS-induced PH in a guinea-pig model by reducing PAP (79). Sildenafil has also been shown to return PAP to normal levels, to attenuate vascular remodelling, and prevent hypoxia-induced PH in a rat model (80). Although the long-term effect of sildenafil on pulmonary haemodynamics in patients with COPD is unknown, treatment with sildenafil significantly increases plasma cGMP levels, reduces PAP increase in healthy volunteers breathing 11% O_2_, and attenuates pulmonary vascular remodelling in mice exposed to hypoxia (81). In addition, sildenafil has been reported to produce significant vasodilation in pulmonary circulation and improve pulmonary haemodynamics by reducing PAP and PVR in patients with COPD-associated PH (82). However, no improvement in exercise capacity has been observed with the use of sildenafil in COPD-related PH (83–85). The apparent absence of a beneficial effect on exercise capacity could be because PDE5 inhibitors possibly worsen gas exchange (84) as consequence of the inhibition of pulmonary hypoxic vasoconstriction (81). Due to the absence of large randomised trials and lack of sufficient evidence, these vasodilator drugs are currently not approved for the treatment of PH in COPD (1).

To minimise the risk associated with the use of systemic vasodilators (e.g., sildenafil) in those with group 3 PH, it has been suggested that inhaled therapies are preferred over oral therapies for this particular group of patients as these drugs can selectively improve perfusion where ventilation is best. Inhaled treprostinil is the first approved therapy for patients with group 3 PH (only due to interstitial lung disease) as it improves exercise capacity (86). Our recent systematic review demonstrated that inhaled therapies targeting prostacyclin pathway can reduce pulmonary vascular resistance without worsening pulmonary gas exchange (87). Although the use inhaled nitric oxide is not currently approved for adults with all forma of PH, limited studies assessed the short-term efficacy of using inhaled nitric oxide in patents with PH due to COPD and demonstrated potential benefits in improving hemodynamic parameters in COPD (88, 89). However, it should be noted that these studies are limited by very small sample size. The findings of the currently ongoing study assessing inhaled sGC stimulator use in PH due to COPD may also provide further evidence on the efficacy and safety of inhaled therapies targeting nitric oxide pathway (78). Given the small number of studies available that have assessed the role of eNOS expression and the release of nitric oxide in the development of COPD-associated PH, the potential usefulness of targeting the nitric oxide pathway, particularly inhaled therapies in the treatment of PH associated with COPD needs further attention. Furthermore, an enhanced understanding of the pathway may lead to a targeted therapy worthy of investigation for the treatment of PH in COPD.

### 3.2. Role of prostanoids and their synthases in COPD-associated pulmonary hypertension

**Figure 1:**
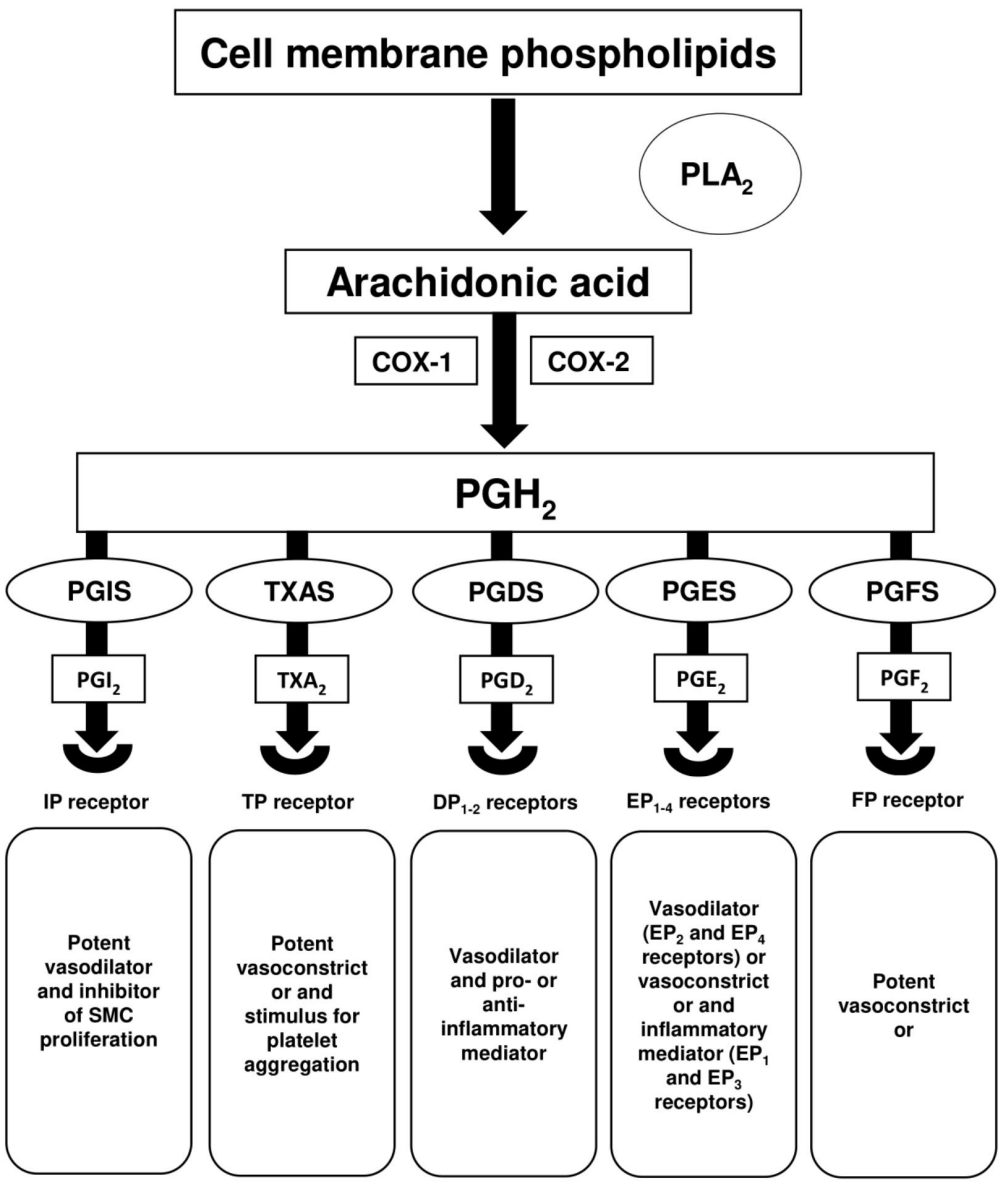
Arachidonic acid metabolism. Hydrolysis of cell membrane phospholipids via phospholipase A_2_ (PLA_2_) releases arachidonic acid (AA), which is then converted by cyclooxygenase-1 (COX-1) and cyclooxygenase-2 (COX-2) to the intermediate prostaglandin H_2_ (PGH_2_). PGH_2_ is converted to multiple prostanoids; prostacyclin (PGI_2_), thromboxane A_2_ (TXA_2_), prostaglandin D_2_ (PGD_2_), prostaglandin E_2_ (PGE_2_), and prostaglandin F_2_ (PGF_2_) via their respective synthases; prostaglandin I synthase (PGIS), thromboxane A synthase (TXAS), prostaglandin D synthase (PGDS), prostaglandin E_2_ synthases (PGESs), and prostaglandin F synthase (PGFS).

Arachidonic acid (AA) and its metabolite pathway play a key role in the homeostasis of vascular smooth muscle and endothelial cells. Abnormalities in this pathway have been shown to be associated with vascular remodelling and PH (90, 91). Vasoactive prostanoids, including prostaglandins (PGs) and thromboxane (TX) are the major metabolites of AA and are formed by COX or prostaglandin G/H synthase. As shown in Figure 1, the synthesis of PGs and TX involves the hydrolysis of cellular phospholipids via the action of phospholipase A_2_ (PLA_2_) enzyme to produce free AA. AA is converted to unstable prostaglandin H_2_ (PGH_2_) by COX activity, and then to main prostanoids PGI_2_, TXA_2_, PGD_2_, PGE_2_ and PGF_2_ via their respective synthases PGIS, thromboxane A synthase (TXAS), prostaglandin D synthase (PGDS), prostaglandin E_2_ synthases (PGESs) and prostaglandin F synthase (PGFS) (92, 93). Currently, there are nine known prostanoid receptors expressed in different cell types, namely IP for PGI_2_, TP for TXA_2_, DP_1-2_ for PGD_2_, EP_1-4_ for PGE_2_, and FP for PGF_2_. Activation of these receptors leads to a wide variety of biological effects, particularly vasodilation or vasoconstriction in vascular cells. IP, EP_2/4_ and DP_1-2_ receptors are considered relaxant receptors, while the activation of contractile receptors (TP, EP_1/3_, and FP) is known to induce vasoconstriction (94).

There are no reports to date showing the involvement of PGF_2_ and PGD_2_ in pulmonary vascular remodelling in all forms of PH. Conversely, PGI_2_, TXA_2_, and PGE_2_ play an important role in maintaining balance in cardiovascular homeostasis (95). Although PGI_2_/TXA_2_ imbalance (96) and PGE_2_ deficiency (97) have been implicated in the development of group 1 PH, studies to date do not provide clear evidence about the role of these vasoactive prostanoids (PGI_2_, TXA_2_, and PGE_2_) in the development of PH in COPD.

#### 3.2.1. COX-2

There are two isoforms of cyclooxygenase (COX): COX-1 and COX-2. COX-1 is expressed constitutively in many cells and serves as a housekeeping gene, while COX-2 is a highly inducible gene, and its expression is increased in response to inflammation (98–101). In addition to the role of COX-2 induction in both inflammation and airway remodelling in COPD (102), it has long been suggested that COX-2 upregulation is involved in pulmonary vascular remodelling (90). Recently, increased COX-2 protein expression has been reported in both vascular endothelial cells in lung tissues of patients with COPD and CS extract-treated human umbilical vein endothelial cells (103). CS extract treatment has also been shown to induce mRNA expression of COX-2 in human pulmonary microvascular endothelial cells (104). Importantly, the COX-2 expression upregulation following CS extract stimulation in human umbilical vein endothelial cells and in patients with COPD is associated with an increased cell apoptosis rate (103). In addition, our recent findings demonstrated that CS extract induced COX-2 in both human PASMCs and PAECs and that COX-2 inhibitor can reduce CS-induced PASMC and PAEC proliferation (60). These findings suggest a role of COX-2 induction in CS-induced pulmonary vascular remodelling in PH.

Besides the effect of CS on COX-2 expression, hypoxia (a key stimulus for vascular remodelling in PH) has also been reported to induce COX-2 expression in human PASMCs (93), in rat lungs (90), PASMCs (93), and in human umbilical vein endothelial cells (105, 106). Although the use of selective COX-2 inhibitor has been shown to increase endothelin-1 release in human PASMCs (107), it has been demonstrated that selective inhibition of COX-2 induction can prevent hypoxic pulmonary vasoconstriction (108), the development of PH in a rat model (109) and chronic hypoxia-induced PH in new-born pigs (110). It has also been reported that the use of selective COX-2 inhibitor can completely block hypoxia-induced proliferation of human umbilical vein endothelial cells (105). These findings suggest the anti-proliferative effect of COX-2 inhibitor in umbilical vein endothelial cells is likely via the inhibition of prostanoid production. Nevertheless, more studies are needed to determine whether the increase in the expression of COX-2 and the production of its downstream vasoactive prostanoid mediators and cell proliferation by hypoxia observed in human umbilical vein endothelial cells exist in human PAECs, the major regulators of vascular function in PH. In addition, COX-2 induction in response to hypoxia in experimental human and animal cellular studies may represent an important mechanism by which aortic smooth muscle cells can increase their capacity for prostanoid production (111).

Collectively, the induction of COX-2 in CS-and hypoxia-treated vascular cells (as well as in the lungs of COPD patients and smokers) suggests a potentially critical role of its downstream vasoactive prostanoid products in vascular remodelling in COPD-associated PH. It is therefore necessary to understand the effect of CS and hypoxia, either individually or in combination, on the expression of COX-2 and its downstream vasoactive prostanoid synthases, and on the release of vasoactive prostanoids in human PASMCs and PAECs, as well as the subsequent impact on cell proliferation and apoptosis.

#### 3.2.2. mPGES-1 and PGE_2_

PGE_2_ is a known inflammatory mediator (112) and plays a role in regulating blood pressure in pulmonary circulation (113). PGE_2_ is mainly formed via the metabolism of AA by COX-1 and COX-2. The free AA is converted into PGE_2_ via COX enzymes and terminal PGESs. Three different types of PGES may be involved in regulating PGE_2_ production: microsomal prostaglandin E synthase-1 (mPGES-1), microsomal prostaglandin E synthase-2 (mPGES-2), and cytosolic PGE_2_ synthase (cPGES). mPGES-2 and cPGES are constitutively expressed in several tissues and are known to have a housekeeping role (114), whereas mPGES-1 is expressed at relatively low basal levels and acts as the terminal enzyme downstream of COX enzymes in producing PGE_2_ from PGH_2_ (115). Amongst the three different types of PGES, studies have shown that mPGES-1 as a key regulator for induced PGE_2_ under inflammatory condition (116–119). Although it is believed that mPGES-1 may have inflammatory effects (120) and deletion of mPGES-1 in the vasculature can be a potential novel target for development of anti-inflammatory drugs (112), the role of mPGES-1 in vascular remodelling in smokers and all forms of PH (including COPD-associated PH) is largely unknown.

PGE_2_ acts on four specific G-protein-coupled receptors in various cell types: EP_1_, EP_2_, EP_3_, and EP_4_. PGE_2_ production in the vascular cells may lead to either vasodilation or vasoconstriction, depending on its binding receptors (115). It is suggested that the stimulation of EP_2_ and EP_4_ receptors could lead to vasodilation by increasing intracellular cyclic adenosine monophosphate (cAMP) concentrations. Vasoconstriction is mediated via the activation of EP_1_ and EP_3_ receptors by increasing intracellular calcium and decreasing intracellular cAMP concentrations, respectively (97). Recently, it has been reported that circulating levels of PGE_2_ are reduced in a chronic hypoxia rat model of PH and in patients with group 1 PH (97). Convincingly, the use of highly selective EP_2_ receptor agonist butaprost has been shown to cause a significant reduction in proliferation of human PASMCs derived from patients with group 1 PH (121). These observations suggest that PGE_2_ can induce a vasodilatory/anti-proliferative effect via EP_2_ in human PASMCs; however, the potential role of PGE_2_ in vascular remodelling in COPD-associated PH has not been studied.

We have previously found that CS extract reduced PGE_2_ levels in human PASMCs extract as a result of the downregulated mPGES-1 expression (60). This suggests that CS-induced a decrease in the levels of PGE_2_ may contribute to PASMC proliferation, leading to pulmonary vascular remodelling in COPD-associated PH. Hypoxia has also been reported to induce PGE_2_ in human PASMCs (93) as well as in human umbilical vein endothelial cells (105). Importantly, exogenous PGE_2_ and hypoxia can stimulate proliferation of human umbilical vein endothelial cells, and this cell proliferation can be inhibited by the combined EP_1/2_ receptor antagonist AH6809 (105). These observations suggest that PGE_2_ may modulate vascular remodelling via EP_1/2_ receptors. However, whether hypoxia affects PGE_2_ production in human PAECs, and whether PGE_2_ can play the same role in hypoxia-induced proliferation of PAECs are both unknown.

Taken together, the role of the mPGES-1/PGE_2_ pathway in vascular abnormalities in PH remains controversial, due to the capability of PGE_2_ and its receptors to either induce or suppress vasodilatory/anti-proliferative and vasoconstrictive/proliferative effects. To date, the effect of hypoxia on mPGES-1 expression and PGE_2_ production in human PASMCs and PAECs, and the potential contribution of PGE_2_ to CS- and hypoxia-induced vascular remodelling have not been determined.

#### 3.2.3. PGIS and PGI_2_

PGI_2_, also called prostacyclin, a potent vasodilator and an inhibitor of PASMC proliferation, was first reported in 1976 by Needleman and Vane (122). PGI_2_ is a downstream product from the sequential enzymatic actions of COX and PGIS and is considered the predominant prostanoid in vasculature. Within the lungs, PGI_2_ is primarily produced in vascular smooth muscle and endothelial cells (123, 124) and is quickly transformed by non-enzymatic procedures to a latent hydrolysis item, 6-keto-PGF_1α_ (125). PGI_2_ acts on the IP receptor to induce vasodilatory and anti-proliferative effects as a result of increased intracellular cAMP concentrations.

In addition to the importance of PGIS/PGI_2_ in group 1 PH, reduced PGIS/PGI_2_ has also been reported in the lungs of smokers with COPD, as well as in CS extract-treated human umbilical vein endothelial cells (126). The study also showed that umbilical vein endothelial cell stimulation with CS extract can induce apoptosis, whilst the use of the PGI_2_ analogue beraprost sodium prevents CS extract-induced cell apoptosis (126). The findings of the same group also demonstrated decreased PGI_2_ production and induced apoptosis in the lungs of CS extract-treated emphysematous rats (127), suggesting that the deficiency of PGI_2_ in CS extract-treated umbilical vein endothelial cells may contribute to cell apoptosis and vascular remodelling. This view is supported by another research group, who showed a downregulation of PGIS mRNA and protein expression and a reduction of PGI_2_ production in lung tissue extracts from patients with emphysema (104). Consistently, we showed that CS extract reduced PGIS mRNA and protein expression and PGI_2_ production in human PASMCs and more importantly, PGI_2_ analogue inhibited CS extract-induced PASMC and PAEC proliferation (60). In primary human pulmonary microvascular endothelial cells, CS extract treatment has also been shown to reduce the mRNA expression of PGIS (104). These observations suggest PGI_2_ may play a crucial role in CS-induced pulmonary vascular cell dysfunction and remodelling in COPD.

Hypoxia has been demonstrated to induce protective effects by further inducing the constitutively expressed PGIS in human aortic smooth muscle cells and human umbilical vein endothelial cells (111), as well as increasing PGI_2_ production in human aortic smooth muscle cells (111) and in human PASMCs (128). Although hypoxia can also increase the mRNA expression of PGIS and the stable metabolite of PGI_2_ in mice chronically exposed to hypoxia (111), increased PGIS expression and PGI_2_ production following the use of PGIS gene transfer has been shown to attenuate medial thickening of small pulmonary arteries and improve hypoxic PH in a mouse model (129). Consistently, PGIS overexpression by gene transfer has been reported to provide protection against the development of hypoxic PH, and to help prevent pulmonary vascular remodelling in a transgenic mouse model (130). In addition, it has been demonstrated that PGI_2_ analogue beraprost sodium can inhibit hypoxia-induced human PASMC proliferation (131). The ability of PASMCs to produce more PGI_2_ following hypoxia exposure, and the role of PGI_2_ analogue in preventing proliferation of PASMCs induced by hypoxia, provide early evidence of the protective effect of PGI_2_ in hypoxia-induced vascular remodelling. However, hypoxia effect on PGIS expression and PGI_2_ production in human PAECs is unknown. In addition, the contribution of hypoxia effect on PGIS/PGI_2_ to the functions of both human PASMCs and PAECs remains to be investigated.

The deficiency of endogenous prostacyclin represents the rationale for targeting the prostacyclin pathway for the treatment of patients with group 1 PH (91). It is suggested that exogenous and endogenous PGI_2_ can induce the relaxation of vascular smooth muscle and inhibit platelet activation (132). Currently, PGI_2_ analogues and PGI_2_ receptor agonists are used as part of the clinical management for treatment of patients with group 1 PH and have been shown to improve exercise capacity, symptoms, and haemodynamics, although the mortality rate associated with group 1 PH has not been significantly reduced with long-term use (133–138). The significant improvements in signs and symptoms of patients with group 1 PH after administration of PGI_2_ analogues and PGI_2_ receptor agonists are likely due to the inhibition of smooth muscle cell proliferation and vasodilating effects on pulmonary arteries (132). However, the efficacy of such treatment in patients with PH in COPD is still unknown due to lack of studies. Recently, the PERFECT trial was conducted to evaluate the effect of inhaled prostacyclin on exercise capacity in patients with PH due to COPD (139). Unfortunately, the PERFECT trial was terminated by the data safety monitoring committee following a routine safety and efficacy analysis (139). Given that the findings of the PERFECT trial have not yet been published, more studies are needed to assess the role of PGI_2_ in COPD-associated PH and the efficacy of PGI_2_ analogues and PGI_2_ receptor agonists use to treat patients with PH due to COPD.

#### 3.2.4. TXAS and TXA_2_

TXA_2_, named after its role in thrombosis, is a potent vasoconstrictor, platelet aggregator, and proliferative mediator with opposing effects to the vasoprotective PGI_2_ in regulating vascular tone (140, 141). Together with PGI_2_, TXA_2_ plays an important role in maintaining homeostatic balance in pulmonary circulation. TXA_2_ is one of the downstream products from COX-2 activity and its production is mediated by the enzyme TXAS. TXA_2_ is unstable and rapidly converted to the inactive metabolite, thromboxane B_2_ (TXB_2_) (142). After being produced by TXAS, TXA_2_ binds to the TP receptor, stimulates the activation of platelet aggregation, and causes pulmonary vasoconstriction as a consequence of intracellular calcium concentration increase (143, 144). Traditionally, platelets were considered the only cellular source of TXA_2_. It is now known that TXA_2_ is produced in a variety of cells including vascular smooth muscle and endothelial cells.

We have previously demonstrated that TXA_2_ is increased in human PASMCs isolated from smokers with COPD compared with TXA_2_ levels in healthy PASMCs (145). More importantly, the balance between vasoconstrictive TXA_2_ and vasoprotective PGI_2_ is found to be important in the homeostasis of vascular function. For example, it has been reported that the balance between these two vasoactive mediators in patients with group 1 PH is shifted away from PGI_2_ toward TXA_2_ (96, 146–148). Although the role Like CS, hypoxia of TXA_2_ in COPD-associated PH is unknown, previous studies suggest CS is a major contributing factor to TXA_2_ increase in COPD. Elevated levels of TXA_2_ have been reported in the urine of smokers and ex-smokers COPD patients, compared with healthy subjects (149). In addition, it has been demonstrated that TXA_2_ is increased in both bronchoalveolar lavage fluid and the lung tissue of rat models exposed to CS (150). Consistent with this animal study, CS extract has been shown to induce TXA_2_ levels in human PAECs (60, 71) as well as PASMCs (60), suggesting that TXA_2_ is a key feature of CS-induced pulmonary artery cell dysfunction.

Like CS, hypoxia can induce pulmonary vasoconstriction and vascular remodelling. However, the involvement of TXA_2_ in this process is not understood. It has been reported that TXA_2_ levels are increased in arterial and venous plasma of hypoxic rats (151). Increased TXA_2_ contributes to pulmonary artery constriction in piglet models of hypoxia-induced PH. Furthermore, the use of a COX-2 inhibitor diminishes the production of downstream COX-2-dependent constrictors, TXA_2_, without adversely affecting other prostanoid production, including PGI_2_ release (110). Concordant with this, using selective COX-2 inhibitors and selective TP receptor antagonists reduces hyperresponsiveness of pulmonary arteries from mice exposed to chronic hypoxia, through blocking TXA_2_-TP receptor signalling (152). In addition, the use of TXAS inhibitor has been shown to blunt the development of hypoxia-induced PH in a neonatal piglet model (153). Although these *in vivo* data suggest that the induction of TXA_2_ production by hypoxia is of critical importance in PH, there are no cellular studies on the effect of hypoxia on TXAS expression and TXA_2_ production in human PAECs and PASMCs. In addition, whether TXAS expression and TXA_2_ production effect plays a role in hypoxia-induced vascular remodelling is largely unknown.

As increased TXA_2_ has been implicated in the pathogenesis of group 1 PH (96), blocking the enhanced TXA_2_ effect could restore PGI_2_/TXA_2_ balance and lead to a therapeutic approach for the treatment of patients with group 1 PH (154, 155). The use of PGI_2_ analogues to compensate for the loss of PGI_2_ production in patients with group 1 PH has been clinically approved and has demonstrated significant improvements in the symptoms of group 1 PH (156). Although early evidence from preclinical animal studies suggests that the inhibition of TXA_2_ by synthase inhibition or receptor antagonism can be effective for hypoxic PH (152, 153), TXAS inhibitor and TXA_2_ receptor antagonist are not clinically approved for the treatment of patients with group 1 PH and other forms of PH, due to lack of any evidence for their efficacy.

Although targeting TXA_2_-TP receptor signalling is not currently used as a therapeutic target for patients with all forms of PH (due to lack of evidence), the inhibition of COX-2-derived TXA_2_ production and blocking TXA_2_ effects using TXAS inhibitors or TP receptor antagonists, have been shown to prevent PH in animal model studies (110, 151–153). Our previous novel findings suggest that blocking increased TXA_2_ effects by the use of TXA_2_ receptor antagonist (daltroban) can exert anti-proliferative effects (60). Furthermore, we showed that the addition of PGI_2_ analogue (beraprost sodium) and the inhibition of CS extract- and hypoxia-induced COX-2-derived TXA_2_ production by COX-2 inhibitor (celecoxib) can restore the balance of prostanoids and help reduce pulmonary vascular remodelling in COPD-associated PH via the inhibition of PASMC and PAEC proliferation. To the best of our knowledge, no clinical trial has been conducted to evaluate the effect of drugs targeting TXA_2_-TP receptor signalling. Thus, a well powered multicentre, randomised, double-blind, placebo-controlled crossover trial to assess the effect of drugs targeting TXA_2_ pathway on clinical outcomes in patients with PH due to COPD is needed.

### 3.3. Endothelin

Endothelin is known as a potent vasoconstrictor and proliferative mediator and was first identified by Yanagisawa and colleagues in 1988 (157). The initial endothelin gene product, prepro-endothelin is cleaved by endopeptidase to pro-endothelin or big endothelin. The big endothelin is subsequently converted into endothelin isoforms via a specific enzyme, called an endothelin-converting enzyme. There are three different isoforms of endothelin: endothelin-1, endothelin-2, and endothelin-3. Endothelin-1 is a 21-amino acid peptide and is found mainly in the cardiovascular system (158). Endothelin-2 differs from endothelin-1 by only two amino acids, shows similar endothelin pharmacology to endothelin-1, and is found primarily in the myocardium, kidney, and placental tissues (159). Endothelin-3 is found mainly in the nervous system, differs by six amino acids from endothelin-1, and is considered a weaker vasoconstrictor when compared with endothelin-1 (159, 160).

Endothelin, latterly named endothelin-1, is the most studied peptide of the endothelin family. Although the primary source of endothelin-1 is considered to be endothelial cells (157), numerous cell types (e.g., PASMCs) can release this peptide *in vitro* upon stimulation with TGF-β1 and pro-inflammatory mediators (161, 162). Once produced, endothelin-1 then binds to either an endothelin-A (ET-A) or endothelin-B (ET-B) receptors (156, 163). ET-B is most often found in vascular endothelial cells, while vascular smooth muscle cells can express both ET-A and ET-B receptors (159). Activation of the ET-B receptor in pulmonary vascular endothelial cells can promote vasodilation by enhancing the release of nitric oxide (146, 164). However, the activation of ET-A and ET-B receptors in vascular smooth muscle cells can lead to vasoconstriction (165, 166).

Elevated endothelin-1 levels in plasma and lung tissues have been shown to be associated with the pathogenesis of groups 1 and 2 PH (167, 168). However, previous studies investigating the role of endothelin-1 on smokers and patients with COPD have been inconsistent and contradictory. It has recently been reported that ET-1 is related to the pathological process of onest as well as development of PH due to COPD (169). Although it has been reported that the levels of endothelin-1 are unchanged in the lung tissue samples of smokers compared with those of non-smokers (69), increased ET-A and ET-B receptors expression has been observed in pulmonary arteries from smokers and COPD patients (170). In addition, an *in vitro* study has shown that CS extract induces endothelin-1 mRNA expression and endothelin-1 secretion in both bovine and human PAECs (171). It also been reported that CS extract induces endothelin release and ET-B receptor protein and mRNA expression in human PAECs (71). The same group has also shown that the use of bosentan (an ET-A and ET-B receptors antagonist) can inhibit CS extract-induced endothelin receptors expression and CS extract-induced proliferation of human PASMCs (170).

In addition to CS, hypoxia plays a key role in the development of PH in COPD (44). However, the contribution of endothelin-1 in this process remains unclear, as results from studies in cultured cells are conflicting. For example, hypoxia induces endothelin-1 mRNA expression in human PAECs (172) and in human pulmonary microvascular endothelial cells (173), whereas hypoxia reduces endothelin-1 production in cultured rat lung endothelial cells (174). Interestingly, CS has been shown to reduce plasma levels of endothelin-1 under hypoxic conditions in guinea pigs, although CS and hypoxia individually have been shown to induce plasma levels of endothelin-1 (61). Thus, there is a need for further *in vitro* investigations to improve our understanding of the effect of CS and hypoxia, either individually or in combination, on the endothelin pathway and the impact of the possible effect in pulmonary vascular remodelling in COPD.

Clinically, the use of bosentan has been shown to foster significant improvement in exercise capacity and haemodynamics in patients with group 1 PH (175–178). This is supported by *in vitro* studies showing that the stimulation of ET-A and ET-B receptors by endothelin-1 treatment in human PASMCs can promote the proliferation of the cells, and may eventually contribute to vascular remodelling and PH (179). Bosentan has also been shown to inhibit the proliferation of human PASMCs from group 1 PH patients (180). These observations suggest that targeting ET-A and ET-B receptors may be a promising therapeutic target for the treatment of group 1 PH. Although the use of bosentan has failed clinically to improve exercise capacity, and hypoxaemia has become progressively worse in COPD without severe PH (181), it has been suggested that the use of bosentan to treat patients with severe or very severe COPD-associated PH can be beneficial (182).

Taken together, evidence from *in vitro* studies showing the inhibitory effect of ET-A and ET-B receptors antagonist on CS extract -induced proliferation of human PASMCs suggests that there may be an important role for the endothelin pathway in CS-induced vascular remodelling in COPD-associated PH. However, the effect of hypoxia with or without CS extract on endothelin release in human PASMCs and PAECs has not been explored. In addition, the contribution of this possible effect to the function of PASMCs and PAECs is yet to be identified.

## 4. Concluding remark

To date, there is currently no treatment approved for patients with COPD-associated PH due to lack of evidence and no proven benefits. While the available evidence suggests that CS and hypoxia, known stimuli of vascular remodelling in COPD, can cause imbalanced vasoactive gene expression and mediator release, the association between pulmonary vascular remodelling and dysregulated prostanoids, nitric oxide and endothelin in PH due to COPD is still not well understood. Given that drugs targeting these three pathways are not currently used for COPD-associated PH and inhaled therapies are preferred over oral therapies to minimise the risk associated with the use of systemic vasodilators in this particular group of patients, more research is urgently needed to assess the safety and efficacy of drugs particularly targeting prostanoids and nitric oxide through inhalation route in patients with COPD-associated PH.

## Author contributions

AAlq: Conceptualization, Supervision, Validation, Writing – original draft, Writing – review & editing. AAld: Conceptualization, Writing – original draft, Writing – review & editing. SAlg: Writing – original draft, Writing – review & editing. JA: Conceptualization, Software, Writing – original draft, Writing – review & editing. RS: Conceptualization, Software, Writing – original draft, Writing – review & editing. HA: Investigation, Software, Visualization, Writing – review & editing. AAlG: Investigation, Software, Visualization, Writing – review & editing. MM: Funding acquisition, Investigation, Software, Visualization, Writing – review & editing. SAls: Investigation, Writing – original draft, Writing – review & editing. LP: Conceptualization, Resources, Supervision, Validation, Writing – original draft, Writing –review & editing.
